# Microarray-Based Analyses of Rhinovirus Species-Specific Antibody Responses in Exacerbated Pediatric Asthma in a German Pediatric Cohort

**DOI:** 10.3390/v14091857

**Published:** 2022-08-24

**Authors:** Erwan Sallard, Katarzyna Niespodziana, Maja Bajic, Thomas Schlederer, Peter Errhalt, Ann-Kathrin Behrendt, Stefan Wirth, Almut Meyer-Bahlburg, Anja Ehrhardt, Rudolf Valenta, Malik Aydin

**Affiliations:** 1 Institute of Virology and Microbiology, Center for Biomedical Education and Research (ZBAF), Department of Human Medicine, Faculty of Health, Witten/Herdecke University, 58453 Witten, Germany; 2 Division of Immunopathology, Department of Pathophysiology and Allergy Research, Center for Pathophysiology, Infectiology and Immunology, Medical University of Vienna, A-1090 Vienna, Austria; 3 Karl Landsteiner University for Health Sciences, 3500 Krems, Austria; 4 Department of Pneumology, University Hospital Krems and Karl Landsteiner University of Health Sciences, 3500 Krems, Austria; 5 Pediatric Rheumatology and Immunology, Department of Pediatrics, University Medicine Greifswald, 17475 Greifswald, Germany; 6 Center for Child and Adolescent Medicine, Center for Clinical and Translational Research (CCTR), Helios University Hospital Wuppertal, Witten/Herdecke University, 42283 Wuppertal, Germany; 7 Laboratory of Experimental Pediatric Pneumology and Allergology, Center for Biomedical Education and Research, School of Life Sciences (ZBAF), Faculty of Health, Witten/Herdecke University, 58455 Witten, Germany

**Keywords:** rhinovirus, RV, virus infection, asthma, wheeze, exacerbation, rhinovirus A, rhinovirus B, rhinovirus C, microarray

## Abstract

Rhinoviruses (RV) account for a significant number of asthma exacerbations, and RV species C may be associated with a severe course in vulnerable patient groups. Despite important evidence on the role of RV reported by clinicians and life scientists, there are still unanswered questions regarding their influence on asthma exacerbation in young patients. Thus, we measured the RVspecies-specific IgG titers in our German pediatric exacerbation cohort using a microarray-based technology. For this approach, human sera of patients with exacerbated asthma and wheeze, as well as healthy control subjects (*n* = 136) were included, and correlation analyses were performed. Concordantly with previously published results, we observed significantly higher cumulative levels of RV species A-specific IgG (*p* = 0.011) and RV-C-specific IgG (*p* = 0.051) in exacerbated asthma group compared to age-matched controls. Moreover, atopic wheezers had increased RV-specific IgG levels for species A (*p* = 0.0011) and species C (*p* = 0.0009) compared to non-atopic wheezers. Hypothesizing that bacterial infection positively correlates with immune memory against RV, we included nasopharyngeal swab results in our analyses and detected limited correlations. Interestingly, the eosinophil blood titer positively correlated with RV-specific IgG levels. With these observations, we add important observations to the existing data regarding exacerbation in pediatric and adolescent medicine. We propose that scientists and clinicians should pay more attention to the relevance of RV species in susceptible pediatric patients.

## 1. Introduction

Asthma is considered as a chronic, heterogenic disease of the airways, and patients with asthma suffer from distinct respiratory symptoms, including cough and dyspnea [1,2,3]. According to the International European Society/American Thoracic Society, severe asthma is defined as a condition where patients require a wide range of several local or systemic medications, including a high dosage of corticosteroids [4]. The beginning of autumn and winter but also spring is mostly associated with an increase in viral infections and in medical consultations [5,6,7,8,9]. In fact, viral infections are a major aggravation factor for children and adolescents with asthma [10,11,12]. Due to the substantial number of asthma exacerbations caused by rhinoviruses (RVs) [13], there are numerous unanswered questions regarding RV influence on the development and course of asthma.

Biologically, RV is ascribed to the family of Picornaviridae and the genus Enterovirus [14,15]. It is a single-stranded, positive sense RNA virus, which has a size of approximately 7200 base pairs [16,17]. The capsid of the RV consists of four proteins, including VP1, 2, 3, and 4, that envelop the genome [16,18]. There are currently more than 160 described RV, which are divided into three phylogenetic species A (80 serotypes), B (32 types), and C (57 types) [19] (https://www.picornaviridae.com/ (accessed on 3 June 2022).

Symptoms of an RV infection are usually self-limiting in healthy individuals without chronic disorders, but individuals with pre-existing diagnoses, including asthma, may endure serious complaints [20]. Patients with RV infection present airway symptoms, e.g., shortness of breath, bronchitis/bronchiolitis, or pneumonia [21,22,23].

Few studies have focused on the importance of RV species in the pediatric population, in particular, in the context of airway infection, wheezing, or asthma [24,25,26,27]. Children with RV-A and -C from the COAST (Childhood Origins of ASThma) cohort suffered from a more severe course [19] compared to those with RV-B who presented a milder clinic [28].

However, there are still less data regarding the impact of RV species A, B, and C on exacerbation in childhood asthma, and the impact of RV on the development and course of the disease is insufficiently understood. In particular, the mechanism through which RV infection affects clinical and molecular markers of childhood asthma, such as atopy, has not been molecularly described in detail. New insights in this field may be of great significance for clinicians and prompt them to rethink the diagnosis and treatment of susceptible patients. Following these open and important questions, we recruited children and adolescents between 3 months and 17 years of age with recurrent obstructive bronchitis and/or asthma suffering from a respiratory exacerbation at a pediatric university hospital in western Germany (including healthy subjects as a control group) and addressed these issues in the biomaterials collected during exacerbation [29,30,31]. In a previous work, we already demonstrated that the majority of children and adolescents were positive for RV/enteroviruses in the nasopharyngeal swab specimens [32]. Measuring antibody levels rather than direct nucleic acid-based strategies for viral genomes could be a better indicator of exposure to virus infections, and the seroprevalence after RV infection is species-specific, as there are more serotypes that are described in the literature. Moreover, the cumulative virus-specific Immunoglobulin (Ig)G responses also inform about past infections, even though individuals may react differentially to similar infections, which makes IgG level an imperfect indicator of infection course (e.g., [33,34,35,36,37,38,39]). With this following work, we focused on the IgG antibody specific levels for the RV species A, B, and C in human sera as a potential predictor of exacerbation and related phenotypes.

## 2. Materials and Methods

### 2.1. Patient Characteristics and Biomaterial Collection

The definition of the group asthma, wheeze, and healthy controls, as well as atopy and steroid ‘phenotypes’, were described previously [29,31,32]. Briefly, the cohorts were stratified according to current diagnosis criteria for asthma, which were set for those who were between five to 17 years of age with respect to the history and lung function parameters (*n* = 49; 9.9 ± 3.3 years, 34 males). Moreover, patients before the age of 5 years suffering from several (at least two) bronchitic/wheezing episodes were defined as wheezers (*n* = 49; 2.1 ± 1.2 years, 33 males). Furthermore, children and adolescents between 3 months to 17 years of age who did not have any chronic disorders and did not have an acute febrile infection within the last weeks were defined as healthy controls (*n* = 38; 8.2 ± 4.5 years, 21 males), and biomaterials were collected, e.g., as part of a diagnostic routine, or other indications). A positive atopic condition was diagnosed when positive information on allergic rhinoconjunctivitis, atopic dermatitis, a blood eosinophilia, and/or a significant positive allergic serology (ImmunoCAP^TM^) were present (asthmatics, *n* = 35 (*n* = 3 atopic not definable); wheezers, *n* = 10 (*n* = 3 atopic not definable), and HC, *n* = 4).

In addition, asthmatics and wheezers who had a history of taking steroids or leukotriene receptor antagonist were classified as steroid-positive subjects. Patients with asthma and wheeze suffering from acute respiratory complains were defined as exacerbated patients (exacerbated asthmatics, exacerbated wheezers) [29,31,32].

During exacerbation (clinical visit), human serum samples (serum sample tubes purchased from Sarstedt AG and Co. KG, Nümbrecht, Germany) and nasopharyngeal swab specimens (asthmatics: *n* = 43, positive culture: *n* = 19; wheezers: *n* = 49, positive culture *n* = 37; healthy controls: *n* = 34, positive culture *n* = 10) were collected [29,30,31,32,40]. The samples of the healthy controls were not recruited during exacerbation; these patients did not suffer from any acute febrile or respiratory symptoms. After collection, serum samples were incubated in dark conditions at room temperature (RT) for approximately 20 min and then centrifuged at 2000× *g* for 15 min, without brake [29]. The serum was carefully portioned and transferred to cryotubes and directly deep-frozen at −80 °C for further experimental studies [29]. For the RV microarray chip measurement, the serum samples used had been already thawed once. The swabs were cultured on an agar plate for up to 48 h at 37 °C, as a typical diagnostic routine, and the bacterial identification was performed using the matrix-assisted laser desorption ionization-time of flight mass spectrometry (MALDI-TOF-MS) [31].

### 2.2. Microarray-Based Determination of Virus-Specific IgG Levels

Thermally oxidized silicon supports diced into rectangles and stuck into aluminum frames (Silicon Valley Microelectronics, Inc., Santa Clara, CA, USA) were coated with an amine-reactive complex organic polymer, MCP-2, (Lucidant Polymers, Sunnyvale, CA, USA) and used for the production of microarrays [41]. Synthetic VP1 N terminal peptides representing three RV genetic species (RV-A: *n* = 18; RV-B: *n* = 9; RV-C: *n* = 10), as well as the recombinant RSV-derived G protein of the A2 strain, were produced and spotted in triplicates using a SciFlexArrayer S12 (Scienion AG, Berlin, Germany) as previously described [42,43].

For the determination of virus-specific antibody responses, microarrays were first washed for 5 min with phosphate-buffered saline with 0.5% Tween 20 (PBST) and dried by centrifugation using a Sigma 2–7 centrifuge and MTP-11113 rotor (both Sigma Laborzentrifugen GmbH, Osterode am Harz, Germany). Subsequently, 30 µL of a 1:300 diluted serum sample (sample dilution buffer was obtained from Thermofisher, Waltham, MA, USA) were applied to each array, and the slides were incubated for 2 h at RT. After another washing step, 30 µL of DyLight 550 (Pierce, Rockford, IL, USA) labeled anti-human IgG (Jackson ImmunoResearch Laboratories, West Grove, PA, USA) were added per array and incubated for 30 min. at RT. After further washing and drying, microarrays were scanned using a confocal laser scanner (Tecan, Männedorf, Switzerland), and the image analysis was performed using the MAPIX software (Innopsys, Carbonne, France). For the calibration and determination of background signals, a calibrator (i.e., Lyphocheck Immunology Plus Control containing 22 serum proteins and related analytes, Bio-Rad Laboratories, Hercules, CA, USA) and a sample diluent were included in each analysis run, respectively. Conversion of the measured fluorescence units to ISAC standardized units (ISU) was performed as previously described [43,44]. The IgG levels considered in the following analyses are, for each species, the average of the measurements for all corresponding antigens of the species present on the chip.

### 2.3. Statistical Analyses

The statistical analysis of the dataset was performed as also described previously [32] using R version 4.2.0 [45] with the packages A Grammar of Data Manipulation (dplyr) [46] and ARTool [47]. The plots were figured with ggplot2 package [48]. The data were analyzed using ART models (non-parametric equivalent of two-ways ANOVA tests), Kruskal–Wallis tests (non-parametric equivalent of one-way ANOVA), or Mann–Whitney U tests in the case of pairwise comparisons and T tests when each group contained enough data for parametric tests (*n* ≥ 30). Linear correlations between two quantitative variables were tested using Pearson’s linear correlation tests. To avoid *p*-hacking, all *p*-values except those from post-hoc pairwise comparisons were corrected using the FDR method. The significance threshold was set at *p* < 0.05.

## 3. Results

### 3.1. Rhinovirus Antibody Levels Are Increased in Exacerbated Asthmatics

**Figure 1:**
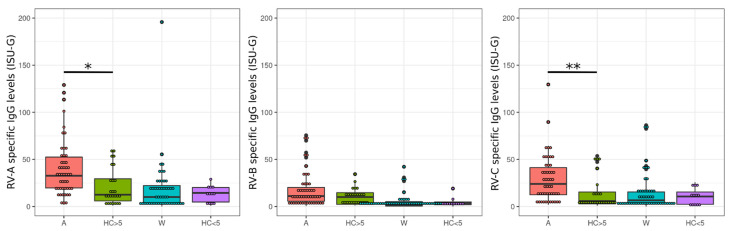
Rhinovirus (RV) species-specific A- and C-IgG levels are higher in asthmatics than in age-matched controls. Exacerbated asthmatics and wheezers were compared with age-matched controls for their species-specific anti-RV-IgG levels, expressed as ISAC standardized units (ISU-G). Results are displayed as box plots, where 50% of the values are within the boxes and non-outliers between the bars. Lines within boxes indicate median values. Here, exacerbated asthmatics revealed higher IgG level of RV-A and -C than healthy controls. The groups were compared using Mann–Whitney-U tests. The *p*-values were corrected using the FDR method. Significant differences were observed between asthmatics and age-matched healthy controls for A species (*p* = 0.011) and C species (*p* = 0.0051). *: 0.01 < *p* < 0.05; **: 0.001 < *p* < 0.01. A: Asthmatics, HC > 5: Healthy controls five years old or older, W: Wheezers, HC < 5: Healthy controls younger than five years old.

Asthmatics are often positive for RV species A and C [49]. In particular, RV species C is reportedly associated with severe symptoms [50]. In our cohort, exacerbated asthmatics demonstrated higher antibody levels for RV species A and C when compared to the healthy control group (Figure 1).

### 3.2. Atopy Strongly Influences RV Antibody Levels

**Figure 2:**
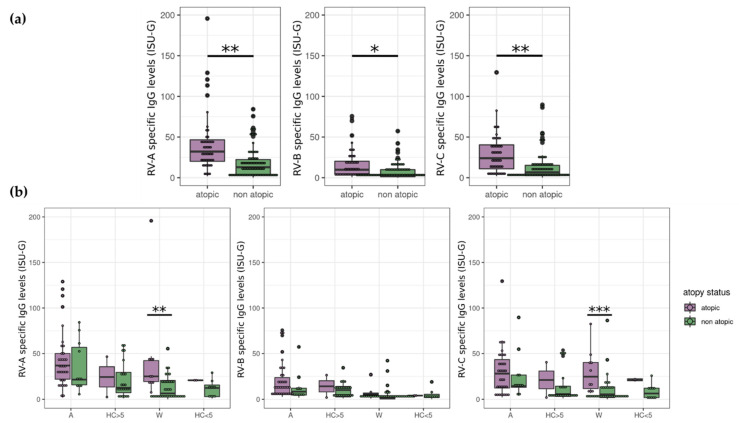
Rhinovirus (RV) species-specific antibody levels are higher in atopic subjects. The extent to which atopic status is associated with RV species was next hypothesized. (**a**) We observed that atopic patients had elevated IgG titers to RV-A, -B, and -C. For each RV species, the virus-specific IgG level of atopic and non-atopic participants was compared using T-tests. *p*-values were corrected using the FDR method. (**b**) Levels of IgG responses to RV-A, -B, and -C species in exacerbated asthmatics, wheezers, and their age-matched controls subdivided according to the atopy status (atopic: Violet; non-atopic: Green). For each species and each age group (older or younger than five years old), the influence on virus-specific IgG level of disease, atopy status, or interaction of both factors were tested using an ART (aligned rank transform) model (non-parametric equivalent of a 2-way ANOVA test). The *p*-values were corrected using the FDR method. In young patients, atopy was associated with higher RV-specific IgG levels of species A (*p* = 0.016) and species C (*p* = 0.030). Post-hoc pairwise comparisons (Mann–Whitney U tests) found significant differences between atopic and non-atopic patients in the wheezers group (*p* = 0.0011 for A-species and *p* = 0.0009 for C-species), but not in young healthy controls. *: 0.01 < *p* < 0.05; **: 0.001 < *p* < 0.01; ***: *p* < 0.001. A: Asthmatics, HC > 5: Healthy controls five years old or older, W: Wheezers, HC < 5: Healthy controls younger than five years old.

Different phenotypes may be associated with a RV species-specific infection and course. In particular, RV species A and C are commonly observed in the context of atopic patients [51]. Thus, we asked if the RV species levels are different within atopic subjects. Here, all atopic patients were included and compared to non-atopics. Importantly, we observed that atopy is significantly associated with high antibody levels against each RV species (*p* = 0.0017, 0.016, and 0.0017 for A-, B-, and C-species, respectively) (Figure 2a). We next searched from which age group(s), if any, the difference between atopics and non-atopics originates. RV-A and -C levels were higher in atopic compared to non-atopic wheezers (*p* = 0.0011 for A-species and *p* = 0.0009 for C-species). This was not the case in atopic/non-atopic asthmatics, where the number of non-atopic asthmatics was also lower or in healthy controls (Figure 2b).

### 3.3. The Steroid Status of Exacerbated Patients Does Not Significantly Correlate with RV-Specific IgG Levels

**Figure 3:**
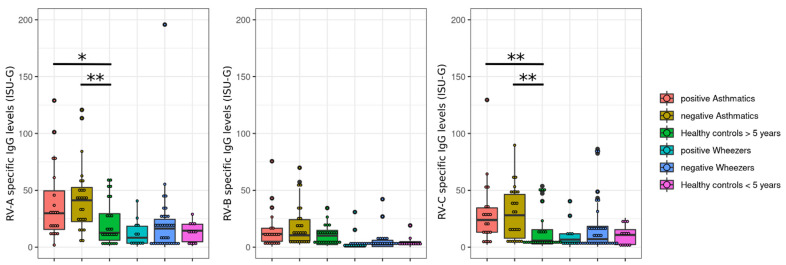
Steroid status does not significantly correlate with RV-specific IgG levels. RV-A, -B, and –C-specific IgG levels are expressed as ISAC standardized units (*y*-axis: ISU-G) in asthmatics and wheezers, grouped according to the intake of corticosteroids or leukotriene receptor antagonist. Asthmatics and wheezers were subdivided between steroid-positive and steroid-negative patients. For each species and each age group, the virus-specific antibody level of steroid-positive patients, steroid-negative patients, and healthy controls were compared using Kruskal–Wallis tests. The *p*-values were corrected using the FDR method. Significant differences were found in participants older than five years old for A- (*p* = 0.030) and C-species (*p* = 0.020). Post-hoc pairwise comparisons (Mann–Whitney U tests) found in each case significant differences between both groups of asthmatics and healthy controls, but no significant differences between steroid-positive and steroid-negative asthmatics or wheezers were found (Positive: Steroid-positive. Negative: Steroid-negative; *: 0.01 < *p* < 0.05; **: 0.001 < *p* < 0.01).

Next, we hypothesized that the intake of corticosteroids locally or systemically or leukotriene receptor antagonists may influence the RV species-specific serum IgG levels. For this, we included the results of steroid-positive and -negative patients (asthmatics and wheezers). Here, we did not observe any significant differences between steroid-positive and -negative exacerbated subjects (Figure 3). However, the levels are lower in steroid-positive subjects.

### 3.4. Nasopharyngeal Test Results Revealed no Correlation between Bacteria and RV

**Figure 4:**
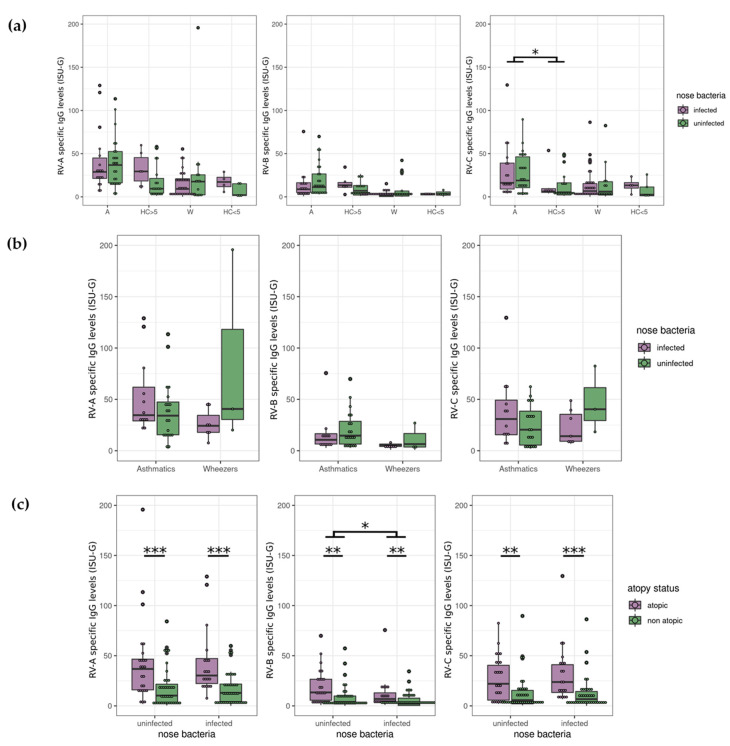
Nose bacteria infection (detection; positive bacterial test result in the nasopharyngeal region) showed variable results within phenotypes. (**a**) For each species and each age group (older or younger than five years old), the influence on RV-specific IgG level of disease, nose bacteria ‘infection’ or interaction of both factors were tested using an ART model (non-parametric equivalent of a 2-way ANOVA test). The *p*-values were corrected using the FDR method. (**b**) Nose bacteria infection does not correlate with RV-specific IgG levels in atopic patients. For each species, the RV-specific IgG levels of nose bacteria infected atopic asthmatics were compared with that of bacteria-uninfected atopic asthmatics using Mann–Whitney U tests. The same comparisons were conducted in atopic wheezers. All *p*-values were corrected using the FDR method. No significant effect of nose bacteria infection on the RV-specific IgG levels of atopic patients was detected for any age group or RV species. (**c**) Participants with nose bacteria infection had a lower RV-specific IgG level of species B. For each RV species, the influence on virus IgG level of atopy status, nose bacteria ‘infection’, or interaction of both factors were tested using an ART model. The *p*-values were corrected using the FDR method. Atopy was significantly associated with higher virus-specific IgG level for all three species (*p* = 0.0000059, *p* = 0.00076 and *p* = 0.000034 for A, B and C-species, respectively). Interaction of atopy and bacteria infection was not significantly correlated with virus-specific antibody levels. Post-hoc pairwise comparisons were conducted using Mann–Whitney U tests (*: 0.01 < *p* < 0.05; **: 0.001 < *p* < 0.01; *** *p* < 0.001; A: Asthmatics, HC > 5: Healthy controls five years old or older, W: Wheezers, HC < 5: Healthy controls younger than five years of age).

Patients with asthma may have increased colonization with distinct bacteria, e.g., *Moraxella*, *Streptococcus*, *Staphylococcus*, and *Haemophilus*, etc., in the nasopharyngeal region as previously described (reviewed in [52,53,54,55]). We asked to what extent bacterial colonization may be RV species-dependent. For this sub-analysis, we used the previously published dataset [31,32], where participants whose nasopharyngeal swabs revealed at least one of the following species: *M. catarrhalis*, *S. pneumoniae*, *S. aureus*, *H. influenzae*, and *H. parainfluenzae*, were classified as ‘bacteria infected’ (representing a culture result). Here, we observe that the ‘infected’ and ‘non-infected’ asthmatics have higher IgG levels against RV species C than the age-matched control group (infected/non-infected), as could be expected from previous results (Figure 1), but did not significantly differ between them (Figure 4a). Excluding the healthy control group from the dataset did not lead to any significant differences between infected and uninfected exacerbated asthmatics or wheezers (Figure 4b). Interestingly, grouping the infected/non-infected patients according to the atopic status, we saw a difference between the uninfected and infected subjects for RV species B (*p* = 0.016), despite it being the RV species showing the least associations with other parameters of asthma phenotype (Figure 4c).

### 3.5. Blood Eosinophils Are Positively Correlated with RV-Specific IgG Levels

**Figure 5:**
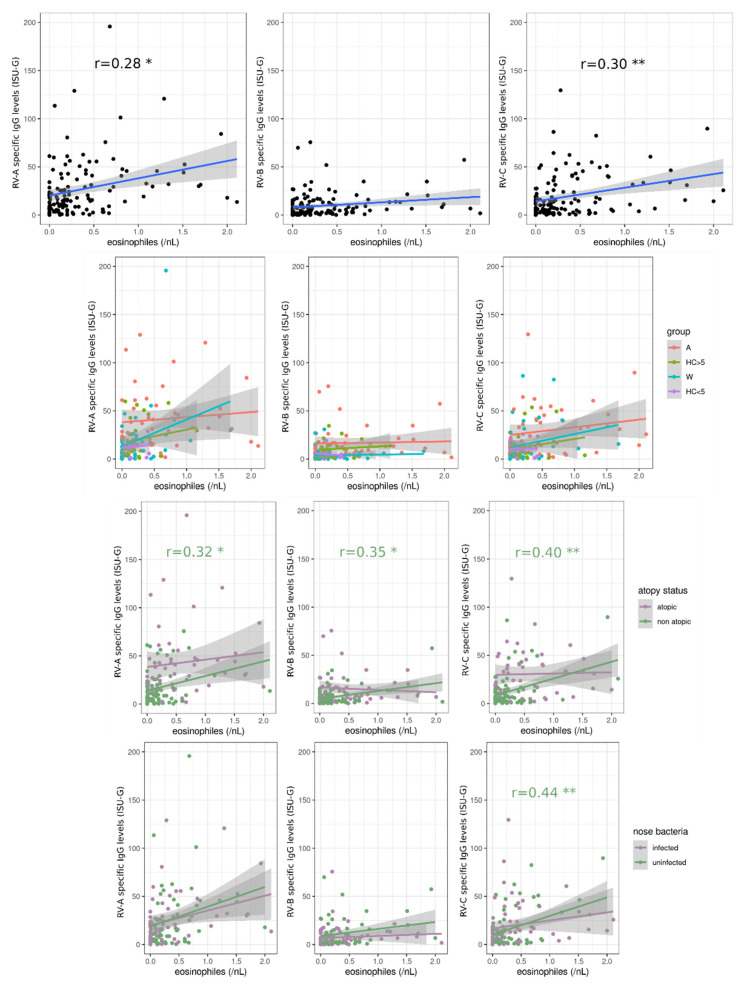
Blood eosinophils are positively correlated with Rhinovirus (RV)-specific IgG levels. For each RV species, the correlation between virus-specific IgG levels and blood eosinophils titer was tested using Pearson’s linear correlation tests, with either group, atopy status, nose bacteria infection status, or no stratification. The correlation coefficient ρ values are shown in the graphs. All *p*-values were corrected using the FDR method. *: 0.01 < *p* < 0.05; **: 0.001 < *p* < 0.01. A: Asthmatics, HC > 5: Healthy controls five years old or older, W: Wheezers, HC < 5: Healthy controls younger than five years old.

In order to determine if immunological parameters play an important role in the RV-specific IgG levels, we next examined the blood eosinophil count of the patients, which is a relevant parameter in those with atopic disorders (reviewed in [56,57]). For this purpose, we also stratified the cohort using other parameters, such as atopy status, age, and positive bacterial test results in the nose. Eosinophils titer was positively correlated with the levels of IgGs specific for RV species A and -C (*p* = 0.016 and 0.010, respectively), and the correlation was surprisingly mainly driven by non-atopic study participants (*p* = 0.030, 0.018, and 0.0067 for A, B and C-species, respectively). Furthermore, we observed that RV species C-IgG level was positively correlated (*p* = 0.0091) with eosinophils in patients without bacterial colonization in the nose (Figure 5).

## 4. Discussion

With this project, we studied IgG levels of RV species in an exacerbated pediatric cohort to understand the clinical aspects influenced by RV or influencing the infection. Due to the plethora of open questions in pediatric asthma, with this pilot work, we aimed to decipher the importance of RV species A, B, and C during acute exacerbation in our cohort. Moreover, the variable presentation of an exacerbation among asthmatics and how the exacerbation in early infancy evokes the progression or even development of asthma are relevant topics.

Asthma exacerbations still continue to be a serious medical problem and are associated with a high hospitalization rate [58,59]. Viruses play a significant role in the development of an exacerbation, where RV constitutes a high proportion of the infections [13]. For many years, scientists have investigated causal aspects of the molecular impact of RV species during host infection and even their influence on disease development [60,61,62,63,64]. Importantly, there are abounding answers and clues on the RV species A, B, and C. In particular, RV species C is known to induce serious clinical events [19,65], e.g., in asthmatics [65,66]. Thus, we investigated through microarray-chip-based technology the RV species-specific IgG levels and observed that the asthma cohort revealed higher IgG levels specific for RV species A and C when compared with healthy controls.

Several studies have investigated the clinical aspects specifically of these two clades in asthmatics. For example, Turunen and co-workers studied the RV species distribution within their pediatric population and revealed positive test results for RV species A and C [51]. Importantly, the authors observed that subjects testing positive for RV species C showed a more serious clinical course associated with more bronchodilator use and having a shorter period of pre-warning symptoms before hospital admission [51].

In the work of Bizzintino and colleagues, the authors presented that not only RV-C was responsible for most exacerbations but also that patients who demonstrated an RV-C-related exacerbation had more severe asthma and high asthma severity scores [65]. This correlation was not observed within our cohort, considering the Asthma Control Test (ACT) and Global Initiative for Asthma (GINA) scores (data not shown). A further study including children with fever or respiratory infections revealed an association between RV-C infection and wheezing, as well as a more severe course [67]. In particular, contrary to RV-A positive patients, affected individuals with RV-C were ventilated and received oxygen [67]. A Taiwanese study confirmed the correlation between RV-C infection and asthma exacerbation and a worse clinic [66]. In addition, Lambert and colleagues reported a sex-dependent course where boys below five years old with RV-C infection were presenting a more moderate/severe asthma course than infected girls [25].

When infants are challenged with an early-onset RV infection, the risk for recurrent wheezing and development of asthma is increasing, and the pathways are still intensely debated in the literature [60,68,69]. In our cohort, we observed that atopic subjects had higher anti-RV IgG levels than those who had a non-atopic phenotype. Moreover, stratifying the subjects into asthma, wheezer, and healthy groups, we saw significant differences between atopic and non-atopic wheezers. Unfortunately, the number of non-atopic asthmatics was low, which prevented us from observing an effect that may have been significant with a larger cohort. Here more non-atopic asthmatics must be recruited to assess this hypothesis. Our results thus may support the works of Turunen and colleagues and Jartti et al., who showed that the atopic status and the illness severity were dependent on the detection of RV-A and -C within their population studies [51,70]. A further study presented that the atopy of the mother was associated with respiratory symptoms of the infants caused by RV [71,72] and that an allergic sensitization of the children was associated with a more severe course [60]. Here, children with wheezing symptoms caused by RV and an aeroallergen sensitization had an increased risk of asthma development [60]. Together with our results, this observation warrants further study on how interactions between both individual and familial atopy, on the one hand, and viral infections, on the other, determine asthma or wheezing exacerbation.

Jartti and colleagues examined the interaction between allergic sensitization and RV infection in children with wheezing [70]. Interestingly, they observed that allergic sensitization was associated only with RV detection in their cohort [70], which also correlates with our findings where RV species A and C were positively correlating with the eosinophil blood titers. Although the authors did not compare the severity of illness between atopic and non-atopic subjects, their study provides a good overview of the relationship between allergic sensitization and RV infection [70]. The hypothesis that allergic sensitization can lead to severe RV infection was proposed by Jackson and co-workers [73]. Accordingly, the authors observed in their study that allergic sensitization was more likely to lead to RV-related wheezing events [73]. However, these studies provide important data in terms of atopy in wheezing and RV infection, and more investigation regarding the relationship between atopic/non-atopic wheezers and RV infection will add valuable information to this field.

Whether our wheezer cohort with an RV infection will progress to asthma will be an intriguing question to follow, as already discussed in our previous work [32]. The classification including transient early, persistent, and late-onset wheezing is currently in use [74] and its presentation is based on and influenced by distinct factors, e.g., perinatal factors, positive family history for atopy, age of mother, pre- and postnatal nicotine abuse [75,76,77,78]. The wheezing phenotype may present important perspectives on the occurrence of asthma [79]. In our previous study, we observed that our cohorts showed a high number of positive test results for RV/enterovirus and respiratory syncytial virus [32]. In our cohort, we did not observe any differences regarding species A, B, and C within the wheezer cohort except for the atopic level. Due to the young age and potentially no or less RV contact, the IgG levels for the RV are low (Figure 1), which may be a physiologic phenomenon (e.g., [80]). Stenberg-Hammer and Niespodziana et al. analyzed the RV species and the changes in the IgG levels at follow-up in young patients (aged 6 months to <4 years) suffering from wheezing symptoms [81]. The authors conclude that more than 70% of wheezing patients entering the emergency ward showed positive test results for RV (in particular for RV species C (74%)), and that 61% of patients had higher RV-specific antibody titers at follow-up visit [81]. Importantly, the increase/change of the IgG levels may also be time-dependent, and the days of reporting symptoms and use of medications also reflect the severity of the symptoms/course [81]. We did not find significant correlations in wheezers between virus-specific IgG level and disease severity as measured by ACT or GINA score (data not shown), but these parameters may not be strong predictors of hospitalization in our opinion, thus explaining the apparent discrepancy between our results and those of Stenberg-Hammer and Niespodziana et al. Unfortunately, due to the smaller number of follow-up visits of our study population, we still lack the data necessary to fully address the question. We plan to continue to follow our cohort or in a new cohort in order to study this phenomenon.

Like in our previous study [32], we found limited correlations between nose bacteria and RV infections and none with the high-concern species A and C. This may suggest that bacterial colonization does not influence RV exposure, which would be contradictory to previous publications (e.g., [82,83,84,85,86,87]), or alternatively, the interactions between pathogens in the nasal cavity are too complex for their influence on asthma to be deciphered on small cohorts. Thus, this will be an important issue to discuss in future studies.

In this article, we focused on serum IgG titers, but PCR detection of RV would provide complementary information on current infections and their relationships with asthma development in real time. Furthermore, PCR analyses would allow to differentiate more than 160 serotypes and thus provide more detailed indications that may be of great use for vaccine development.

In this study, we included those serum results from subjects who had a prior nasopharyngeal swab testing for virus analyses via BioFire^®^ Film Array^®^ Respiratory 2.1 plus panel (Biomerieux). Regardless of which test result (positive or negative) was present in the healthy control cohort, we still interpreted this cohort as ‘healthy’ because the patients showed no evidence of acute infection either in the history or clinical examination. In contrast to the diseased cohorts, asthmatics and wheezers were suffering from acute respiratory symptoms, including cough, rhinitis, shortness of breath, chest tightness, etc. However, it remains to be discussed whether a positive virus test result must correlate with current asthma symptoms, as asthma is associated with a multifactorial etiology. Of course, since PCR tests for RV genome detection were not performed, a false-positive result cannot be excluded. Of note, the study was performed in only one part of Germany, so over-regional analyses and comparisons with other centers in the same country are currently not presented. A further limitation factor of this study is that there is no ‘biological’ information about the course of our cohorts. This means we have no information on the phase before exacerbation and no longitudinal data after exacerbation, and therefore, appropriate comparative analyses cannot currently be performed. Although we collected the ACT/GINA data as part of our questionnaire and obtained clinical information about the past weeks, we have not performed biomaterial collection at these respective time points. Thus, an analysis of asthmatics and/or wheezers between current and previous infection, as well as current infection and longitudinal course, are not possible, which would significantly improve the data interpretation. Despite these limitations, it should nevertheless be taken into account that the biomaterial recruitment of exacerbated children and adolescents is difficult to obtain and that many of our results confirm and extend previously published data [32].

Taken together, our pediatric exacerbation cohort demonstrated high IgG levels for RV species A and C. This adds to the already available evidence that these can lead to asthma exacerbation and points out the need to amplify the efforts in vaccine development. Although similar results are already published in the literature, e.g., [49], with our cohort, we add complementary observation to the field of pediatric exacerbation, especially in Germany, where only a small number of data are still presented. Further functional experiments must be performed to delineate the corresponding pathomolecular relevance of these species in distinct wheezing and asthma endo- and phenotypes.

## Data Availability

The data presented in this study are available on request from the corresponding authors.
